# Applications of node-based resilience graph theoretic framework to clustering autism spectrum disorders phenotypes

**DOI:** 10.1007/s41109-018-0093-0

**Published:** 2018-08-29

**Authors:** John Matta, Junya Zhao, Gunes Ercal, Tayo Obafemi-Ajayi

**Affiliations:** 1 Department of Computer Science, Southern Illinois University Edwardsville, Edwardsville, IL USA; 2 Department of Computer Science, Missouri State University, Springfield, MO USA; 3 Engineering Program, Missouri State University, Springfield, MO USA

**Keywords:** Graph theory, Clustering, Autism spectrum disorders, Resilience measures

## Abstract

With the growing ubiquity of data in network form, clustering in the context of a network, represented as a graph, has become increasingly important. Clustering is a very useful data exploratory machine learning tool that allows us to make better sense of heterogeneous data by grouping data with similar attributes based on some criteria. This paper investigates the application of a novel graph theoretic clustering method, Node-Based Resilience clustering (NBR-Clust), to address the heterogeneity of Autism Spectrum Disorder (ASD) and identify meaningful subgroups. The hypothesis is that analysis of these subgroups would reveal relevant biomarkers that would provide a better understanding of ASD phenotypic heterogeneity useful for further ASD studies. We address appropriate graph constructions suited for representing the ASD phenotype data. The sample population is drawn from a very large rigorous dataset: Simons Simplex Collection (SSC). Analysis of the results performed using graph quality measures, internal cluster validation measures, and clinical analysis outcome demonstrate the potential usefulness of resilience measure clustering for biomedical datasets. We also conduct feature extraction analysis to characterize relevant biomarkers that delineate the resulting subgroups. The optimal results obtained favored predominantly a 5-cluster configuration.

**Electronic supplementary material:**

The online version of this article (10.1007/s41109-018-0093-0) contains supplementary material, which is available to authorized users.

## Introduction

Clustering comprises a prolific research area for data exploration and knowledge discovery applications with a great variety of approaches. With the growing ubiquity of data in network form, clustering in the context of a network represented as a graph has become increasingly important. In graph theory contexts, clustering involves finding a k-partitioning of the vertices of a graph. The concepts and properties of graph theory make it very convenient to describe clustering problems by means of graphs (Xu and Wunsch II 2009). Nodes *V*={*v*_*i*_,*i*=1,…,*N*} of a weighted graph *G* correspond to *N* data points in the pattern space, and edges *E*={*e*_*ij*_,*i*,*j*∈*V*,*i*≠*j*} reflect the proximities between each pair of data points. Use of graph theoretic clustering techniques is not restricted to cases where the data is inherently graph-based. They have also been shown to be effective on other types of data by transforming the data to a graph form using an appropriate graph representation (Alpert et al. 1999). Brugere et al. (2018) provide an in depth overview regarding creating networks from data as well as examples of network structure inference in diverse fields such as computational biology, neuroscience, epidemiology, ecology, and mobile device technology.

There are many benefits to converting data to a network representation as networks are an excellent way of representing complex relationships. The following benefits are highlighted and discussed in details in Ref. (Brugere et al. 2018). Networks aid in uncovering the higher-order structure emerging from dyadic relationships. They are also useful in exploring the heterogeneity that exists among individual entities. Diverse measures can be applied in interpreting and/or evaluating network representations such as density, degree distribution, clustering coefficient, centralities, etc. Networks are interpretable models for further analysis and hypothesis generation. Many useful tools also exist for network analysis that can be used across domains. Thus, networks provide a common language through which biological researchers can communicate with computer scientists. Graph based methods aid ease of visualization of analysis, a natural co-occurrence of network representation. Given a dataset, the main challenge usually lies in determining which particular network will be the most useful representation to provide meaningful inference.

There are various successful examples of the use of graphs in analyzing biological and health-related data. Pan et al. (2018) converted gene expression data to an appropriate graph representation and computed *betweenness centrality* (a graph-theoretic measure) to find important regulator genes in tumors. Their study is a useful motivation for the current work, which also uses betweenness centrality in a heuristic to find important data points. Dale et al. (2018) employed graph clustering techniques to gene expression data to identify genes potentially related to powdery mildew disease resistance in grapevines. Alves et al. (2018) applied graph clustering and graph theoretic measures (degree distribution, average clustering coefficient, and average short path length) to evaluate the effects of an antibody on chick embryos. The specific application of classification of human traits and diseases in patient networks using graph analysis is conducted for a variety of medical applications including pathological narcissism in (Pierro et al. 2018), dark personality traits in (Marcus et al. 2018), post-traumatic stress disorder in (Akiki et al. 2018), and inflammatory bowel diseases in (Abbas et al. 2018).

This paper investigates the application of graph theoretic clustering on analysis of clinical data relating to Autism Spectrum Disorder (ASD) phenotypes. Clinical data, such as in ASD, is commonly characterized by significant heterogeneity, high dimensionality, complexity in structure and mixture of variables, disparate data sources, and missing data. There is a critical need to identify and validate more homogeneous subgroups as well as learn the distinct features (biomarkers) associated with the subgroups. This work significantly extends preliminary results presented in (Matta et al. 2017) on clustering ASD phenotype data using our *node-based resilience* clustering framework (NBR-Clust) (Matta et al. 2016; Borwey et al. 2015). NBR-Clust is unique in its focus on critical attack sets of *nodes* *S*⊂*V* whose removal disconnects the network into multiple components that form the basis of resultant clusters. Due to natural properties of sparse node-cuts, the NBR-Clust approach is useful not only for traditional clustering scenarios where the number of clusters may be unknown a priori, but also for clustering in the presence of outliers or noise, and/or overlapping nodes (Matta et al. 2016; Borwey et al. 2015). In (Matta et al. 2016), we generalized the usefulness of node-based resilience measures for clustering, particularly when the number of clusters is not known a priori. We conducted an in-depth comparative analysis using existing known resilience measures such as integrity, toughness, tenacity, and scattering number as well as a parametrized version of vertex attack tolerance (VAT). The results obtained demonstrated the effectiveness of VAT and integrity over the other methods in clustering the datasets with high accuracy. Additionally, integrity was likely to cluster datasets in one step, and tenacity was useful for giving an upper bound to cluster number determination.

In this work, we conduct a systematic exploration of application of NBR measures to delineate heterogeneous ASD data into more meaningful subgroups using a sample population drawn from the Simons Simplex Collection (Fischbach and Lord 2010). We investigate three NBR measures (VAT, Integrity and Tenacity) along with multiple graph constructions to determine appropriate representations for the ASD phenotype data. We also employ feature extraction techniques to determine a potential set of ASD phenotype biomarkers that discriminate the resulting subgroups. A varied set of statistical methods is applied to validate and interpret the clinical significance of the results.

## Autism spectrum disorders

ASDs are childhood neurodevelopmental disorders diagnosed on the basis of behavioral assessments of social, communicative, and repetitive symptoms (Association et al. 2013). Although ASD is behaviorally distinctive and reliably identified by experienced clinicians, it is clinically and genetically extremely heterogeneous (Miles 2011). Children with ASD exhibit a wide diversity in type, number, and severity of social deficits, behaviors, and communicative and cognitive difficulties, which are assumed to reflect multiple etiologic origins (Eaves et al. 1994). Given the increase in ASD prevalence (Autism and Developmental Disabilities Monitoring Network Surveillance Year 2010 Principal Investigators 2014) and the corresponding increasing associated economic burden (Lavelle et al. 2014), there is a need for automated approaches to detect more homogeneous subgroups of patients, and more importantly for biomarkers (biologically based phenotypes) to inform tailored intervention and improved outcomes. Biomarkers are useful to index diagnostic status or risk, demonstrate engagement of specific biological systems, and provide more rapid assessment of change than traditional measures based on clinical observation and caregiver report (McPartland 2016). In the unsupervised learning context, biomarkers can be regarded as significant features that characterize a subgroup (or cluster). Thus, the problem of inferring meaningful biomarkers translates to unsupervised learning of discriminant features. A better understanding of heterogeneity in autism itself, based on scientifically rigorous approaches centered on systematic evaluation of the clinical and research utility of the phenotypic and genotypic markers (Georgiades et al. 2013), would generate useful information for the study of etiology, diagnosis, treatment and prognosis of the disorder.

There have been varied cluster analysis approaches on ASD phenotype/clinical data over the past two decades. Prior to DSM-5 (Association et al. 2013), some of these approaches (Stevens et al. 2000; Ingram et al. 2008; Cuccaro et al. 2012) focused on exploring empirical subgroups that aligned with pre-defined subgroups (such as ASD DSM-IV subtypes) or illuminated some knowledge on etiologically distinct subgroups i.e. which behavioral and physical phenotypes will most likely subdivide ASD. Since the introduction of the DSM-5, emphasis is placed on the spectrum of autism i.e. on a severity gradient under the diagnostic umbrella of Autism Spectrum Disorder. According to Georgiades et al. (2013), the task of categorizing the clinical heterogeneity in children with autism is still of critical importance, regardless of how the DSM changes its definition. Hence, there have been even more studies (Georgiades et al. 2013; Ousley and Cermak 2014; Veatch et al. 2014; Obafemi-Ajayi et al. 2015; Al-Jabery et al. 2016; Nguyen et al. 2018) that attempt to better classify the ASD heterogeneity under DSM-5 using a varied set of ASD phenotype data. Some ASD studies (Chaste et al. 2015) suggest that attempts to stratify children based on phenotype will not increase the power of ASD genetic discovery studies. This is possibly true when the methods are limited by a very restricted set of phenotyping variables (diagnosis, IQ, age at first words, ASD severity, insistence sameness, and symptom profiles) and do not account for possible outliers in the dataset. Spencer et al. (2018) demonstrated that ASD phenotype subgroups could aid discovery of novel ASD genes. It is important to employ clustering methods that simultaneously identify and remove possible outliers that could be skewing the results and add pertinent and relevant phenotype ingredients that may uncover meaningful subtypes. Ultimately, the validity of any subgrouping paradigm depends on whether the ASD subgroups actually uncover/expose some biologic or genetic variation, which can be used to predict prognosis, recurrence risks or treatment responses. Hence, in this work, we also apply rigorous statistical analysis to validate the significance of the results as well as guide the optimal clustering configuration selection.

## Clustering framework

### NBR-Clust Algorithm

Node-based resilience measures compute a critical attack set of nodes *S*⊂*V* whose removal disconnects the network with relative severity. Given a node-based resilience measure, NBR-Clust conducts robust clustering by using the set of components that result from the removal of the computed critical attack set as a basis for the set of clusters. We explore the following three node-based resilience measures in this work: vertex attack tolerance (VAT), integrity, and tenacity.

The VAT of an undirected, connected graph G = (V, E) is denoted *τ*(*G*) and defined as (Ercal 2014; Matta et al. 2017)

1 $$ \tau(G) = \min_{S \subset V, S \neq \emptyset} \left\{\frac{|S|}{|V-S-C_{max}(V-S)|+1} \right\}  $$

where *S* is an attack set and *C*_*max*_(*V*−*S*) is the largest connected component in *V*−*S*.

Normalized integrity (Barefoot et al. 1987) is defined as

2 $$ I(G) = \min_{S \subset V} \left\{ \frac{|S| + C_{max}(V-S)}{|V|} \right\}.  $$

Tenacity (Cozzens et al. 1995) is defined as

3 $$ T(G) = \min_{S \subset V}\left\{\frac{|S|+C_{max}(V-S)}{\omega (V-S)}\right\},  $$

where *ω*(*V*−*S*) is the number of connected components in *V*−*S*.

Traditional clustering usually ensures assignment of all nodes to a specific cluster. In complex datasets, some nodes could be outliers (nodes that don’t really belong to a specific cluster) or overlapping nodes (i.e. nodes that could be assigned to more than one cluster). In these scenarios, the critical attack set may be used to determine outliers or overlap data points(Matta et al. 2016; Borwey et al. 2015). In this work, we consider both the traditional complete clustering scenario where all critical attack nodes are reassigned to cluster-components, as well as the non-traditional situation where the critical attack set is removed from the base clusters (i.e. without node reassignment). Given that we are clustering phenotype data that could involve some errors from the data collection process, outliers would imply potential erroneous data points. Removal of these outliers may result in better defined clusters. Overlap nodes could also be a pertinent feature, like in biological networks when proteins are classified to different clusters to reflect their multiple functions. However, the concept of overlap nodes is not clearly defined for medical data. We plan to explore this concept further in future work.

The NBR-Clust algorithm consists of four main phases:

i) Transform point data into a graph G;
ii) Approximate resilience measure of graph, R(G), with acceptable accuracy, and return the candidate attack set S whose removal results in some number of candidate groupings (components C);
iii) Perform a node-assignment strategy that assigns each node of S to a component C from step ii;
iv) If more clusters are desired, choose the component with the lowest resilience measure and divide it into additional components using steps ii and iii. If fewer clusters are desired, join components with the greatest number of adjacent edges. The dividing and combining can continue until a desired number of clusters is obtained.

The VAT-Clust, Integrity-Clust, and Tenacity-Clust algorithms (Borwey et al. 2015; Matta et al. 2016) utilize a heuristic known as Greedy-betweenness centrality (Greedy-BC). The *betweenness centrality* of a node is the ratio of shortest paths that include that node to the total number of shortest paths. High betweenness centrality is a measure of the importance of a node, as it implies that the node is more likely to be part of a path used when traversing the graph. The Greedy-BC heuristic estimates candidate attack sets by repeatedly taking the highest betweenness node, removing it from the network, taking the next highest betweenness node, removing it from the network, etc. Matta (2017); Matta et al. (2017) demonstrated that Greedy-BC approximates VAT, integrity and tenacity with acceptable accuracy. We implemented the NBR-Clust framework using weighted betweenness centrality computations (Brandes 2001).

In the NBR-Clust method, if there is a desired number of clusters *k* for the output clustering configuration, a regrouping or hierarchical (Borwey et al. 2015) algorithm can be applied to attain this. None of the three clustering algorithms are guaranteed to output an exact *k* number of clusters. When more clusters are produced than desired, we regroup clusters by finding the pair of current components C1 and C2 that maximizes the normalized cut quantity: E(C1,C2)/(C1*C2), where E(C1,C2) is the number of edges between C1 and C2 and C1*C2 is the product of the number of nodes in C1 and the number of nodes in C2. C1 and C2 are combined into one cluster. Regrouping of clusters is repeated until the desired number of clusters is obtained. If the algorithm outputs fewer clusters than desired, then the hierarchical approach (Borwey et al. 2015) is applied to split the clusters till the specified number of clusters is achieved.

### Data preprocessing

Given that the sample was drawn from a rigorous data collection (Simons Simplex Collection (Fischbach and Lord 2010), it contained very few missing values, approximately 0.1% missing values. Majority of the missing values were localized in two features, out of a total of 36 features. To impute missing values for these two attributes we used a standard regression, computed in Matlab, on the remaining 34 attributes to determine likely values. For other features that had very few missing values (0.002*%*), the mean of the remaining values for the specific feature was used.

Feature selection is commonly used for selecting a small subset of features for building a learning model with good generalization performance (Guyon and Elisseeff 2003). Usually, the task of a feature selection algorithm is to prune the feature space by eliminating as many irrelevant and redundant features as possible and thus reducing the dimensionality of the dataset. In the dataset used, the number of features is relatively small compared to the number of examples. We apply the correlation filter algorithm introduced in (Obafemi-Ajayi et al. 2017) to exclude highly correlated features from the subsequent analysis. The filter algorithm automatically identifies and filters highly correlated features using pairwise Pearson correlation function based on a user defined threshold value. In this work, we investigate the effect of applying the correlation filter prior to clustering vs. simply using the entire set of features.

### Graph representations

To apply the NBR-Clust framework on our dataset, we first convert the data into a k-nearest neighbor (kNN) graph *G*. In a kNN graph *G*_*k*_, vertices *u* and *v* have an edge between them if *v* is amongst the *k* closest vertices to *u* with respect to the distance metric considered. While any distance metric may be used to determine nearness of neighbors, we use the *n*-dimensional Euclidean distance following normalization of the feature space, where *n* is the number of features considered.

In both (Matta et al. 2016; Cukierski and Foran 2008) evidence is presented in favor of minimal connectivity (min-conn) parameter *k* in the construction of kNN graph *G*_*k*_. Min-conn *k* implies choosing the minimal *k* such that $\phantom {\dot {i}\!}\: \forall \;k' \ge k \; \forall _{(u, v \in V)} \; \exists \text { \textit {u-v} path in} G_{k'}$. Additional information may be revealed at different levels of connectivity. A graph where parameter *k* is above connectivity contains more information in the form of additional edges. If nodes that should be clustered together are near to each other, edges are more likely to be added within potential clusters than between them. This will make it easier to identify clusters, and may give better clustering results than graphs where *k* is at minimum connectivity. The cost of using additional information is increased time and complexity.

We consider three different connectivity settings for *k* in the kNN graph construction: min-conn, min-conn+1, and min-conn+2.

### Determining optimal clustering configuration

We applied a holistic approach in determining the most optimal set of results per resilience measure (VAT, Integrity, and Tenacity) using three main criteria: internal cluster validation indices (ICVIs), graph quality measures, and distribution of resulting clusters. Clustering configurations that resulted in clusters with very few nodes (i.e. less than 10) were discarded, given that we had a total of 2680 nodes to cluster. Highly un-skewed clustering configurations tend to bias the cluster validation indices.

An internal cluster validation index determines the optimal clustering solution most appropriate for the input dataset based on two measurement criteria: Compactness and Separateness (Kovács et al. 2005). Compactness measures how close the members of each cluster are to each other. Separateness measures how separated the clusters are from each other. The optimal cluster configuration should yield clusters that are compact and well separated. We explored the application of nine commonly used ICVIs (Liu et al. 2010, 2013; Aggarwal and Reddy 2013) (Silhouette index (SI), Davies-Bouldin (DB) index, Dunn’s index, Xie-Beni index (XB), Calinski-Harabasz (CH) index, I index (I), SD validity index (SD), S_Dbw validity index (S_Dbw), and Clustering Validation index based on Nearest Neighbors (CVNN)) on the clustering results to measure the goodness of the clusters. The metrics are described fully in (Liu et al. 2010, 2013) and were implemented following their guidelines. We applied a large number of ICVIs to attain a more robust decision, given multiple studies (Brun et al. 2007; Vendramin et al. 2010; Arbelaitz et al. 2013; Liu et al. 2013) that demonstrate the diversity in range of results chosen by different indices. The optimal number of clusters is determined based on the majority vote of the validation indices along with the graph validation measures. A summary of these internal validation metrics utilized in this work for selecting the optimal clustering configuration is presented in Table 1. The notations and definitions employed are similar to those presented in (Liu et al. 2013).

**Table 1:** Summary of internal cluster validation used to determine optimal clustering configuration

Validation Metric	Mathematical Description	Optimal Value
Silhouette index (SI)	$\frac {1}{k}{\sum \nolimits }_{i}^{}\bigg \{\frac {1}{n_{i}}{\sum \nolimits }_{x \in C_{i}}^{}\frac {b(x)-a(x)}{\max _{x}[b(x),a(x)]}\bigg \}$ where	Max
	$a(x) = \frac {1}{n_{i} - 1} {\sum \nolimits }_{y \in c_{i}, y \neq x} d(x,y)$ and	
	$b(x) = min_{j, j\neq i}\bigg [ \frac {1}{n_{j}} {\sum \nolimits }_{y \in C_{j}} d(x,y) \bigg ]$	
Calinski-Harabasz index (CH)	$\frac {{\sum \nolimits }_{i}^{}n_{i}d^{2}(c_{i},c)/(k-1)}{{\sum \nolimits }_{i}^{}{\sum \nolimits }_{x \in C_{i}}^{}d^{2}(x,c_{i})/(N-k)}$	Max
Davies-Bouldin index (DB)	$\frac {1}{k}\sum _{i}\max _{j, j \neq i} \bigg [ \frac {\frac {1}{n_{i}}\sum _{x \in C_{i}}d(x,c_{i}) + \frac {1}{n_{j}}\sum _{x \in C_{j}}d(x, c_{j})}{d(c_{i},c_{j})}\bigg ]$	Min
Dunn’s index	$\min _{i}\bigg [\min _{j}\frac {\min _{x \in C_{i}, y \in C_{j}}d(x,y)}{\max _{k} \{\max _{x,y \in C_{k}} d(x,y) \}}\bigg ]$	Max
Xie-Beni index (XB)	$\frac {\sum _{i}\sum _{x \in C_{i}}d^{2}(x,c_{i})} { N \min _{i,j \neq i}d^{2}(c_{i},c_{j})}$	Min
SD validity index (SD)	*Dis*(*k*_*max*_)*Scat*(*k*)+*Dis*(*k*) where	Min
	$Scat(k) = \frac {1}{k}\sum _{i}||\sigma (C_{i})|| / ||\sigma (D)||$ and	
	$Dis(k) = \frac {\max _{i,j}d(c_{i},c_{j})}{\min _{i,j}d(c_{i},c_{j})}\sum _{i}\Big [\sum _{j}d(c_{i},c_{j})\Big ]^{-1}$	
S_Dbw validity Index (SD_Dbw)	*Scat*(*k*)+*Dens*_*bw*(*k*) where	Min
	$Dens\_bw(k) = \frac {1}{k(k-1)} \sum \limits _{i} \bigg [ \sum \limits _{j, j \neq i} \frac {\sum \limits _{x \in C_{i} \cup C_{j}}f(x, u_{ij})}{max \big \{ \sum \limits _{x \in C_{i}}f(x,c_{i}),\sum \limits _{x \in C_{j}}f(x,c_{j}) \big \}} \bigg ]$	
*I* index	$\bigg [\frac {\sum _{x \in D}d(x,c)}{k \sum _{i}\sum _{x \in C_{i}}d(x,c_{i})}\max _{i,j}d(c_{i},c_{j})\bigg ]^{p}$	Max
CVNN index	$ \frac {Sep(k,NN)}{\max \limits _{k}SEP(k,NN)} + \frac {Com(k)}{\max \limits _{k}Com(k)} $ where	Min
	$Com(k) = \sum _{i}\Big [\frac {2}{n_{i}(n_{i}-1)}\sum \limits _{x,y \in C_{i}}d(x,y)\Big ]$ and	
	$Sep(k,NN) = \max _{i}(\frac {1}{n_{i}}\sum _{j}^{n_{i}}\frac {q_{j}}{NN})$ where	
	*O*_*j*_ is the jth object in *C*_*i*_, and *q*_*j*_ is the number of nearest neighbors	
	of *O*_*j*_ which are not in cluster *C*_*i*_.	

*D* denote the data set; *N*: number of objects in *D*; *C*: center of *D*;

*k*: number of clusters; *C*_*i*_: the i–th cluster; *n*_*i*_: number of objects in *C*_*i*_;

*c*_*i*_: center of *C*_*i*_; *d*(*x*,*y*): distance between *x* and *y*; NN : number of nearest neighbors

Since the clustering is done on graph representations of the data, we also utilized specific graph quality measures to evaluate the quality of the resulting graphs: modularity (Newman 2006) and conductance (Arora et al. 2009).

1. **Modularity**: This quantifies the strength of *modules* (analogous to clusters) created when clustering a graph. A graph with high modularity has more than expected edges internal to its modules, and fewer than expected edges between modules. We applied modularity to evaluate the “clusterability" of a graph based on a minimal threshold of 0.6.
2. **Conductance**: The conductance of a cluster is the fraction of all edges in the graph that point outside the cluster (Yang and Leskovec 2012). A low conductance implies a “better” cluster, because a higher proportion of a graph’s edges are internal to that cluster. For our experiments, clustering configurations were acceptable conductance-wise if they had a conductance value of 0.07 or less.

### Feature Extraction Phase

The objective of this phase is to obtain a set of features that discriminate among the clusters, as these features could be potential biomarkers for delineating the ASD subgroups. We employed the BestFirst search method (Eibe et al. 2016), implemented in Weka (Hall et al. 2009). The BestFirst search method traverses the attribute (feature) space to find a good subset. The quality of the subset found is measured by an attribute subset evaluator. It performs a greedy hill climbing, i.e. searching forward from the empty set of attributes, toward the goal of finding the most locally predictive attributes. The CFS (Correlation-based Feature Selection) subset evaluator was used to determine the merit of each subset. The CFS subset evaluator (Frank et al. 2016) assesses the predictive ability of each attribute individually and the degree of redundancy among them, preferring sets of attributes that are highly correlated with the class but with low inter-correlation.

## ASD phenotype data

### Description of phenotype features

The ASD sample analyzed in this work is drawn from the Simons Simplex Collection (SSC) (Fischbach and Lord 2010) population, a comprehensive, rigorous, reliable and consistent dataset supported by the Simons Foundation for Autism Research Initiatives (SFARI). (Simplex indicates that only one child in the family is affected with ASD while both parents and at least one sibling are unaffected.) To ensure reliability of clustering results, individuals missing any Autism Diagnostic Interview-Revised (ADI-R) (Lord et al. 1994) or Autism Diagnostic Observation Schedule (ADOS) (Lord et al. 1989) scores were excluded. The final dataset consisted of 2680 subjects, 2316 males (86.4%) and 364 females (13.6%) between ages of 4 and 17 years old.

In cluster analysis, the quality of input features has a significant impact on the outcome. Hence, having a robust and diverse set of features is key to meaningful results. In contrast to previous work (Nguyen et al. 2018; Matta et al. 2017; Al-Jabery et al. 2016; Obafemi-Ajayi et al. 2015), we included some new sets of features: ADOS social affect score, word delay, ADI-R Q86 abnormality evident score, and ADI-R Q30 language total score. A total of 36 features (Table 2) were used in this work that spanned core diagnostic (ADIR and ADOS scores), ASD-specific symptoms, cognitive and adaptive functioning (IQ score), language and communication profiles (Vineland adaptive measures and Social Responsiveness Scale (SRS) scores, regression, and word delay), behavioral problems (Aberrant Behavior Checklist (ABC), Repetitive Behavior Scale (RBS), and Child Behavior Checklist (CBCL) scores), and possible genetic indicators (Parents’ Broader Autism Phenotype Questionnaire (BAPQ) scores).

**Table 2:** Description of 36 phenotype features used to cluster ASD sample

Category	ASD phenotype features
*ASD-specific symptom scores*	ADOS communication & social interaction score
	ADOS restricted & repetitive behavior score
	ADOS Social Affect score
	Social score (ADI-R A)
	Verbal score (ADI-R B)
	Repetitive and stereotyped patterns of behavior (ADI-R C)
	Abnormality evidence (ADI-R Q86)
*Cognitive & Adaptive functions*	Vineland social score
	Vineland daily living skills score
	Verbal & non-verbal IQ score
*Language & Communication*	Vineland communication score
	Regression
	Word delay
	Overall Level of Language (ADI-R Q30)
*Behavioral problems*	ABC ^*a*^ aggregate scores (stereotype, lethargy, irritability, hyperactivity, inappropriate speech)
	RBS ^*b*^ aggregate scores (compulsive, self-injurious, stereotyped, ritualistic, restricted, and sameness behavior)
	CBCL ^*c*^ internalizing and externalizing problems T scores
	SRS ^*d*^ parent aggregate scores (awareness, cognition, communication, mannerisms, motivation)
	SRS ^*d*^ parent T score
*Genetic indicators*	BAPQ ^*e*^ mean overall scores (Father & Mother)

^a^ABC: Aberrant Behavior Checklist;

^b^RBS: Repetitive Behavior Scale

^c^CBCL: Child Behavior Checklist;

^d^SRS: Social Responsiveness Scale.

^e^BAPQ: Broader Autism Phenotype Questionnaire

All experimental analysis involving human subjects were carried out under the guidelines and approval of Missouri State University Institutional Review Board.

### Statistical analysis of ASD outcome measures

Additional features, not used in clustering, were selected as outcome measures to assess the clinical relevance of resulting cluster configuration. These include overall (total) scores for ABC, RBS, IQ, Vineland II composite standard score as well as the ADOS calculated severity score (ADOS CSS), a history of non-febrile seizures (i.e. diagnosis of epilepsy), and Peabody Picture Vocabulary Test (PPVT-4A) standard score. Note that these outcome measures are not completely independent of the input features used for clustering. We included the total scores of each of the aggregate features (ABC, RBS, IQ, Vineland) applied in the cluster analysis, as these scores tend to provide an overall picture of the ASD severity level of the proband. For example, the Vineland composite score provides an overall picture of the adaptive functioning skills. The ADOS CSS is a quantitative variable calculated using the summation of the ADOS social communication and RRBs scores. It provides a continuous measure of overall ASD symptom severity that is less influenced by child characteristics, such as age and language skills, than raw totals (Hus et al. 2014). It can be used to compare ASD symptom severity across individuals of different developmental levels. As such, they provide a "purer" metric of overall ASD severity. A higher level implies higher severity with 10 as the highest level of severity. The PPVT-4A score quantifies the language skill. A higher score implies fewer deficits, and better developed skills. The epilepsy data was only available for 99.85% of the sample.

To validate the significance of the differences (quantified by mean and standard deviation) in these outcome measures by clusters, we employed the univariate one-way analysis of variance (ANOVA) test along with the Tukey HSD test (pairwise comparisons) for continuous variables (all except epilepsy). The ANOVA p-value reported for each ASD measure generalizes the Student’s t test for between comparisons for multiple groups. The Tukey test informs us on which pairs of clusters are actually statistically different since the ANOVA’s p value only indicates that at least one cluster is statistically different from another. The eta squared test (*η*^2^) was conducted to determine the overall effect size for each clustering configuration per feature. The effect size conveys the practical significance of the ANOVA results. The Cohen’s d test was also applied to quantify the effect sizes for each pairwise comparison.

## Evaluation results and analysis

### Experimental setup

In the evaluation of our model, we investigate the effect of the following parameters:

- NBR measure: VAT, Integrity and Tenacity algorithms were employed with the NBR-Clust framework.
- Critical attack set (*S*): we compared the performance of reassignment of all nodes belonging to *S* (i.e. complete clustering) to no node reassignment of *S*.
- Connectivity level of the kNN graph representation: from minimum connectivity (kNN2) to two above connectivity (kNN4).
- Use of correlation filter algorithm: the threshold value was set at 0.8. This resulted in removal of three features (ADOS Social Affect, Verbal IQ, and SRS T score). We compared the performance using the entire set of 36 features to clustering with only these 33 features (tagged as “corr” in the results).
- Number of clusters (*k*): Based on prior work on subgrouping of ASD patients (Ingram et al. 2008; Cuccaro et al. 2012; Georgiades et al. 2013; Ousley and Cermak 2014; Veatch et al. 2014; Obafemi-Ajayi et al. 2015, b; Al-Jabery et al. 2016; Nguyen et al. 2018), we varied the number of clusters from *k*=2 to 5. The determination of the optimal number of clusters was independent of our NBR-Clust framework but rather based on what is reported in ASD literature and also from previous DSM-IV subtypes (Lord et al. 2012).

Each feature was normalized between 0 and 1 using known standard score ranges for the phenotype feature. The source code of the NBR-Clust algorithm is publicly available at (Node-Based Resilience Measure Clustering Project Website 2018) while the cluster validation platform suite is accessible at (Nguyen and Obafemi-Ajayi 2017). The statistical analyses were implemented using IBM SPSS software while the feature extraction experiments were carried out in WEKA(Frank et al. 2016).

The combinations of different levels of connectivity (kNN2, kNN3, kNN4) and using all features (36) versus correlation filtered set (33) resulted in a total of six base graphs. These six graphs were clustered using VAT-Clust, Integrity-Clust, and Tenacity-Clust, to yield results that had *k*=2, 3, 4 and 5 clusters with and without attack set node reassignment for a total of 144 different clustering output configurations.

### Results

The critical attack set node reassignment results (traditonal clustering) are analyzed separately from without node reassignment (NR) configurations. The set of 7 optimal clustering configurations selected based on majority voting scheme of the nine ICVIs and graph quality measures per NBR measure algorithm is presented in Table 3. The instances where the clustering output attained the best score for the specified ICVI or graph quality measure are highlighted in bold. All optimal configurations, except for Tenacity-Clust with node reassignment, were obtained from the kNN2 graphs, implying the usefulness of min-connectivity graphs, as expected. Four out of the seven groupings examined in Table 3 favored a 5-cluster configuration as optimal. In general, the filtered data set did not seem to demonstrate an impact on the clustering outcomes, except in the case of the kNN3 graphs.

**Table 3:** Optimal Cluster configuration by graph type and resilience measures

	Complete clustering				No node reassignment		
	Integrity k=3	Tenacity k=5	VAT k=4	kNN3 Integrity ^*a*^k=5	VAT k=2	Integrity k=5	Tenacity k=5
Silhouette *↑*	0.11	0.05	0.07	0.07	0.12	0.04	0.05
Davies-Bouldin *↓*	3.18	4.28	4.19	4.40	3.37	3.66	3.75
Xie-Beni *↓*	3.38	7.16	8.10	8.92	3.01	5.77	6.48
Dunn *↑*	0.13	0.15	0.15	0.17	0.14	0.14	0.14
Calinski-Harabasz *↑*	152.57	154.11	166.22	165.32	167.71	142.52	141.58
I Index *↑*	0.14	0.08	0.12	0.08	0.12	0.06	0.09
SD Index *↓*	9.96	14.62	14.40	20.10	8.52	7.71	9.10
SDb w Index *↓*	1.37	1.07	1.16	1.06	1.87	1.10	1.05
CVNN Index *↓*	1.38	0.95	1.21	0.54	2.00	2.00	2.00
Separability *↑*	31.63	11.14	20.21	8.34	8.53	11.39	15.25
Modularity (> 0.6)	0.42	0.72	0.65	0.68	0.27	0.67	0.68
Conductance (< 0.07)	0.02	0.04	0.03	0.06	0.06	0.05	0.04

^a^kNN3 using Integrity measure on correlation filtered data

**Fig. 1:**
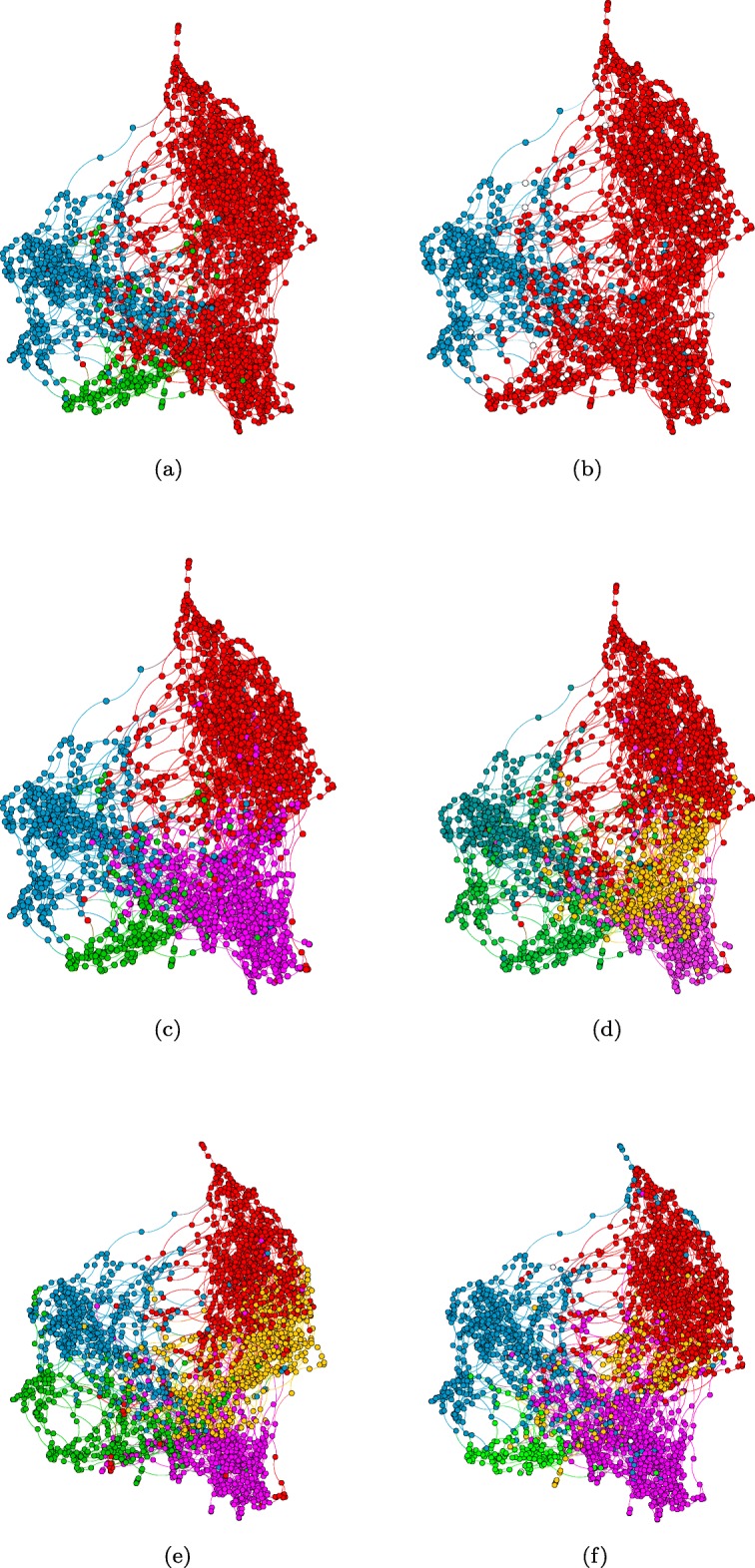
Visualization of optimal clustering results by resilience measure. NR indicates no reassignment of attack set nodes. **a** kNN2 Integrity k=3. Red nodes denotes C0, Blue: C2, and Green: C1. **b** kNN2 VAT k=2 NR. Red nodes denotes C0, and Blue: C1. **c** kNN2 VAT k=4. Red nodes de- notes C2, Blue: C3, Purple: C0, and Green: C1. **d** kNN2 Integrity k=5 NR. Red nodes denotes C0, Blue: C2, Gold: C1, Purple: C3, and Green: C4. **e** kNN2 Tenacity k=5. Red nodes denotes C2, Blue: C3, Gold: C0, Purple: C1, and Green: C4. **f** kNN2 Tenacity k=5 NR. Red nodes denotes C1, Blue: C2, Gold: C3, Purple: C0, and Green: C4

**Fig. 2:**
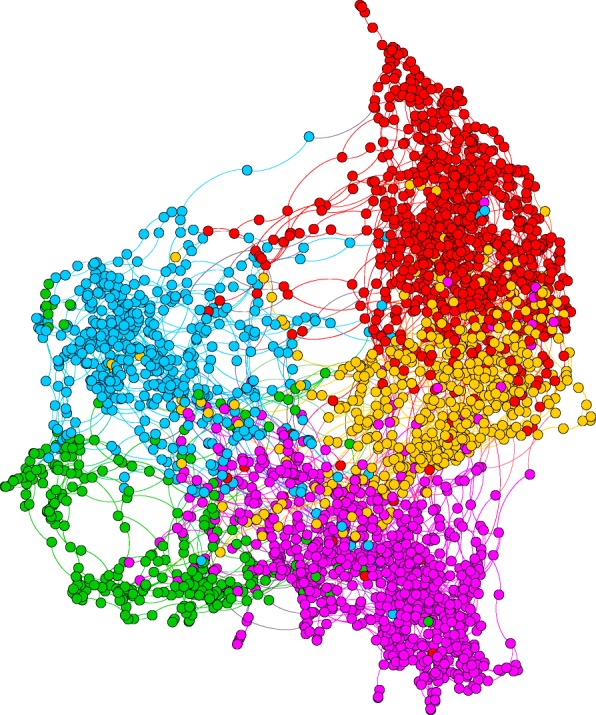
Visualization of the graph of k=5 optimal clustering result for kNN3 with Integrity using the correlated filtered set. Red nodes denotes C2, Blue: C4, Gold: C0, Purple: C3, and Green: C1

The visualizations of the set of 7 optimal clustering configurations, using the ForceAtlas layout algorithm in Gephi (Bastian et al. 2009), are illustrated in Figs. 1 and 2.

The demographics (mean age at ADOS, ethnicity as quantified by percentage Caucasian, and gender) of each cluster of the optimal clustering configurations are shown in Tables 4 and 5. We observe that there are no significant differences in the demographics across clusters for age and gender distribution. However, the distribution of percentage Caucasian varied across clusters.

**Table 4:** Demographics per cluster configuration with node reassignment

	Integrity k=3			VAT k=4				Tenacity k=5				
	C0	C1	C2	C0	C1	C2	C3	C0	C1	C2	C3	C4
Mean age	9.0	8.4	8.5	8.9	8.6	8.9	8.6	9.0	9.0	8.9	8.6	8.6
% Caucasian	81.0	64.5	73.3	78.3	64.4	83.9	71.5	82.6	77.5	83.7	71.90	69.3
% Male	86.4	84.1	87.2	87.2	84.9	85.8	87.2	85.4	89.7	86.6	87.4	82.0

**Table 5:** Demographics per cluster configuration without node reassignment

	kNN2 Tenacity k=5						kNN2 Integrity k=5						kNN2 VAT k=2		
	C0	C1	C2	C3	C4	S	C0	C1	C2	C3	C4	S	C0	C1	S
Mean age	8.8	9.0	8.7	8.9	8.6	9.32	8.9	8.8	8.7	9.3	8.4	9.6	8.9	8.6	8.6
% Caucasian	78.7	85.3	71.1	74.5	64.5	77.8	83.8	77.4	71.6	79.3	67.2	83.3	79.9	71.7	74.5
% Male	88.3	85.3	86.3	86.1	84.9	100.0	86.0	85.1	87.3	88.5	85.4	94.4	86.1	87.2	90.2

Statistical analyses of the optimal clustering configurations for each ASD outcome measures are presented in Tables 6 and 7. Note that for the no node reassignment results (Table 7), though the mean and standard deviation values for *S* is reported for each outcome measure, it is excluded from the Anova, Tukey and Eta-squared analysis. Higher values of ADOS CSS, RBS, ABC and SRS scores implies greater ASD severity levels while higher values of full scale IQ, Vineland composite, and PPVT 4A scores implies lesser ASD severity levels. (The cohen effect size pairwise comparison results are included as a Additional file 1). We can observe that the overall effect sizes, as quantified by the *η*^2^ value is consistently high for kNN2 Tenacity 5-Cluster result in Table 6. Cluster C4 appears to be the most severe ASD subgrouping in terms of low overall IQ, relatively high occurrence epilepsy (non-febrile seizures), low functioning skills (as quantified by the Vineland composite scores), and high ADOS CSS scores. However, their ABC and RBS-R scores are not the most severe scores, and are slightly better compared to cluster C0. Cluster C0 has very high mean IQ scores (not the highest - C2), but the ABC and RBS-R scores for that subgroup are the lowest. For the no node reassignment analysis (Table 7), the 2-cluster VAT-clust result does not seem to convey much practical significance based on the relatively low *η*^2^ values across all ASD outcome measures evaluated.

**Table 6:** Statistical analysis of optimal clustering configurations (complete clustering) by graph type and node resilience measure using selected ASD outcome measures

Cluster (size)	ABC overall	RBS R overall	ADOS CSS	Vineland composite score	Overall IQ	PPVTA 4A	Epilepsy
kNN 2 Integrity k=3							
C0 (1903)	45.73(25.8)	27.31(18.1)	7.37(1.7)	75.72(10.9)	88.19(23.5)	91.51(24.7)	1.74%
C1 (189)	54.08(24.6)	29.01(13.8)	7.58(1.5)	57.57(9.6)	38.91(18.7)	41.75(22.3)	6.91%
C2 (588)	47.55(25.9)	26.26(16.3)	7.63(1.6)	70.58(12.3)	69.99(27.6)	72.41(28.8)	2.73%
ANOVA *p*-value	< 0.001	0.15	0.003	< 0.001	< 0.001	< 0.001	
Tukey HSD (NS *‡*)	C0:C2	All pairs	C1:C0,C2	None	None	None	
Eta-squared (*η*^2^)	0.007	0.001	0.004	0.157	0.244	0.209	
kNN 2 VAT k=4							
C0 (811)	50.86 (28.0)	31.76 (19.6)	7.48 (1.7)	72.69 (10.3)	80.06 (22.8)	82.98 (24.1)	2.47%
C1 (219)	60.02 (28.5)	32.33 (17.4)	7.68 (1.5)	57.74 (9.4)	39.55 (18.7)	41.93 (21.3)	5.99%
C2 (1117)	41.55 (22.2)	23.89 (15.4)	7.24 (1.7)	78.16 (10.4)	94.77 (20.8)	98.34 (22.1)	1.25%
C3 (535)	45.73 (25.2)	25.08 (16.0)	7.70 (1.6)	70.50 (12.8)	69.12 (28.2)	71.39 (29.1)	2.82%
ANOVA *p-value*	< 0.001	< 0.001	< 0.001	< 0.001	< 0.001	< 0.001	
Tukey HSD (NS^a^)	None	C0:C1;C2:C3	C0:C1,C3 C1:C3	None	None	None	
Eta-squared (*η*^2^)	0.047	0.046	0.013	0.212	0.322	0.288	
kNN 2 Tenacity k=5							
C0 (535)	67.12(21.5)	41.78(18.2)	7.30(1.7)	73.71(9.7)	91.46(21.6)	96.46 (23.8)	1.31%
C1 (497)	36.57(19.6)	22.10(12.6)	7.40(1.6)	74.56(10.2)	80.65(22.5)	82.57 (23.4)	2.42%
C2 (781)	33.78(19.3)	18.34(11.4)	7.22(1.7)	78.85(11.0)	92.99(22.8)	96.59(22.9)	1.41%
C3 (484)	44.05(23.9)	25.35(15.5)	7.58(1.7)	74.29(11.0)	79.31(23.7)	81.04(24.4)	2.07%
C4 (383)	61.19(27.5)	33.83(18.9)	7.97(1.5)	58.62(9.5)	42.20(19.4)	44.86(22.8)	5.76%
ANOVA *p-value*	< 0.001	< 0.001	< 0.001	< 0.001	< 0.001	< 0.001	
Tukey HSD (NS *‡*)	C1:C2	none	C0:C1,C2,C3 C1:C2,C3	C0:C1,C3 C1:C3	C0:C2;C1:C3	C0:C2;C1:C3	
Eta-squared (*η*^2^)	0.274	0.253	0.022	0.272	0.361	0.333	
kNN 3 Integrity k=5 corr							
C0 (462)	66.56(24.0)	41.65(18.3)	7.54(1.7)	73.26(9.6)	87.95(22.9)	92.57(24.5)	0.87%
C1 (276)	57.20(27.4)	29.89(15.5)	7.39(1.4)	57.38(8.9)	37.27(17.8)	39.84(21.8)	5.82%
C2 (743)	33.48(18.7)	17.39(10.6)	7.18(1.7)	79.86(10.6)	96.22(20.6)	99.49(21.6)	1.62%
C3 (744)	46.25(24.8)	28.80(17.8)	7.53(1.6)	72.53(10.4)	78.72(23.3)	80.97(24.9)	3.10%
C4 (455)	42.58(23.2)	24.26(14.9)	7.66(1.8)	73.63(12.0)	77.54(25.2)	79.01(26.2)	1.54%
ANOVA *p-value*	< 0.001	< 0.001	< 0.001	< 0.001	< 0.001	< 0.001	
Tukey HSD (NS *‡*)	C3:C4	C1:C3	C0:C1,C3,C4 C1:C2,C3,C4 C3:C4	C0:C3,C4 C3:C4	C3:C4	C3:C4	
Eta-squared (*η*^2^)	0.197	0.215	0.011	0.258	0.354	0.306	

^a^NS: implies pairs for which Tukey HSD test was not significant

The mean and standard deviation values are presented for each measure

**Table 7:** Statistical analysis of optimal clustering configurations using selected ASD outcome measures: for kNN2 graphs without node reassignment

Cluster (size)	ABC overall	RBS R overall	ADOS CSS	Vineland composite score	Overall IQ	PPVTA 4A	Epilepsy
kNN 2 VAT k=2							
C0 (2072)	47.01(26.1)	27.74(17.9)	7.38(1.7)	73.99(12.0)	83.50(27.1)	87.51(27.9)	2.27%
C1 (506)	45.66(25.3)	25.01(16.1)	7.69(1.6)	70.47(12.9)	69.09(28.5)	71.46(29.3)	2.77%
S (102)	46.03(21.6)	26.89(15.1)	7.52(1.5)	73.71(9.6)	81.75(23.2)	77.94(35.1)	0.98%
ANOVA *p-value*	0.295	0.002	< 0.001	< 0.001	< 0.001	< 0.001	
Eta-squared (*η*^2^)	0	0.004	0.005	0.013	0.042	0.049	
kNN 2 Integrity k=5							
C0 (1096)	41.02(22.0)	23.30(14.8)	7.19(1.7)	78.36(10.3)	94.63(20.6)	97.91(22.2)	1.37%
C1 (430)	67.76(24.6)	43.94(19.2)	7.61(1.7)	70.22(9.7)	78.58(23.8)	83.02(25.0)	1.86%
C2 (402)	41.49(23.8)	24.42(15.8)	7.57(1.7)	73.88(11.3)	78.34(24.3)	79.36(25.4)	2.00%
C3 (384)	32.54(18.1)	18.99(10.5)	7.47(1.6)	75.41(10.6)	82.90(21.9)	84.73(23.3)	2.09%
C4 (350)	59.67(27.2)	30.74(17.2)	7.83(1.5)	58.58(9.9)	40.55(19.3)	43.70(22.9)	6.03%
S (18)	56.33(22.6)	32.50(15.0)	7.72(1.9)	69.33(14.2)	68.78(36.4)	65.56(46.7)	11.1%
ANOVA *p-value*	< 0.001	< 0.001	< 0.001	< 0.001	< 0.001	< 0.001	
Tukey HSD (NS^a^)	C0:C2	C0:C2	C1:C2,C3,C4; C2:C3,C4	C2:C3	C1:C2	C1:C2,C3	
Eta-squared (*η*^2^)	0.211	0.210	0.019	0.279	0.383	0.329	
kNN 2 Tenacity k=5							
C0 (741)	47.01(26.1)	29.38(18.8)	7.46(1.6)	73.25(9.9)	81.26(22.4)	83.63(23.3)	2.30%
C1 (951)	41.23(22.4)	22.84(14.8)	7.08(1.7)	78.68(10.5)	95.58(20.3)	99.41(21.6)	1.26%
C2 (591)	45.06(26.3)	25.66(17.3)	7.61(1.7)	71.44(13.3)	70.86(28.5)	72.95(29.9)	2.54%
C3 (216)	68.07(25.8)	41.92(17.0)	8.56(1.5)	68.76(8.9)	75.79(27.1)	80.39(28.1)	2.33%
C4 (172)	54.72(25.2)	28.90(14.0)	7.35(1.4)	56.13(8.9)	36.17(16.6)	37.64(18.6)	7.60%
S (9)	46.22(19.4)	23.56(15.7)	7.78(1.0)	72.67(11.8)	79.00(23.5)	81.88(34.3)	0%
ANOVA *p-value*	< 0.001	< 0.001	< 0.001	< 0.001	< 0.001	< 0.001	
Tukey HSD (NS *‡*)	C0:C2	C4:C0,C2	C0:C2,C4 C4:C1,C2	None	C2:C3	C0:C3	
Eta-squared (*η*^2^)	0.078	0.086	0.054	0.214	0.298	0.270	

^a^NS: implies pairs for which Tukey HSD test was not significant. S is not included in the ANOVA, Tukey, and Eta-squared analyses

The mean and standard deviation values are presented for each measure)

**Fig. 3:**
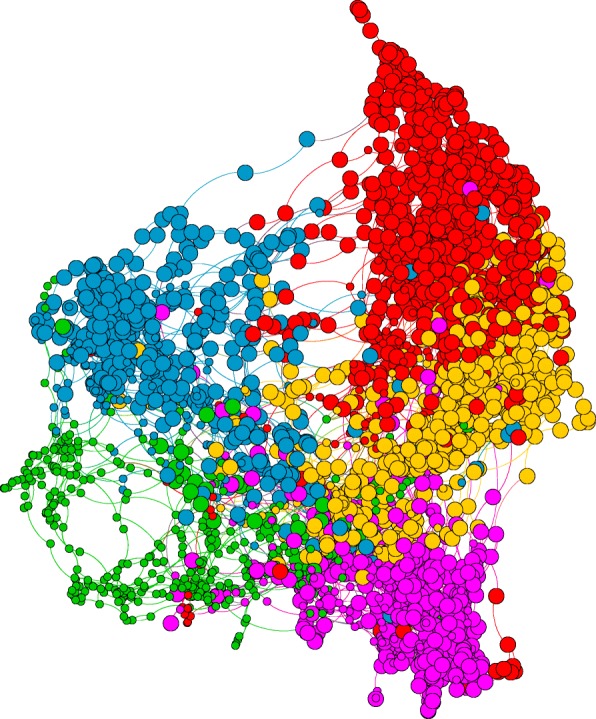
Visualization of optimal clustering result for kNN2 Tenacity 5-cluster graph in terms of distribution of high overall IQ (≥ 70) vs. lower IQ (< 70). Large circles denote high IQ while small circles denote low IQ. Only green cluster shows a high concentration of low IQ nodes. This demonstrates that the clustering obtained is a combination of various factors, not just IQ scores

Figure 3 illustrates the visualization of the graph of the optimal clustering result for kNN2 Tenacity 5-Cluster results in terms of distribution of high overall IQ (≥ 70) vs. lower IQ (< 70). Large circles denote high IQ while small circles denote low IQ. Only the green cluster (C4) shows a high concentration of low IQ nodes (small circles). We can observe the complexity of the variation in the 5-cluster result given by Tenacity kNN2 with node reassignment. This demonstrates that the resulting clustering obtained is a combination of various factors, not just IQ scores.

The outcome of the feature extraction phase is summarized in Tables 8 and 9 for each of the seven clustering configurations. Overall, 20 different features were uncovered as discriminant for at least one of the 7 optimal clusterings. The regression feature was consistently selected for all seven results. Overall level of language (ADI-R Q30) was selected six times while both BAPQ Mother overall average score and word delay were selected five times.

**Table 8:** Set of discriminant features by clustering result for complete clustering configuration

Integrity k=3	Tenacity (corr) k=5	VAT k=4	kNN3 Integrity k=5
ADI-R Q30 (Overall level of language)	ADI-R Q30 (Overall level of language)	ADI-R Q30 (Overall level of language)	ADI-R Q30 (Overall level of language)
ADI-R Q86 (Abnormality evidence)	RBS-R (Ritualistic Behavior)	ADI-R Q86 (Abnormality evidence)	ABC-Inappropriate speech
CBCL Externalizing T Score	ABC-Irritability	Verbal score (ADI-R B)	RBS-R-Stereotyped behavior
Regression	BAPQ Avg (Mother)	BAPQ Avg (Mother)	BAPQ Avg (Mother)
	Regression	Regression	Regression
	ADOS Social Affect	ADI-R C (Repetitive behavior)	ADI-R C (Repetitive behavior)
	Social (ADI-R A)	SRS Mannerisms	Social (ADI-R A)
	SRS T score	Word delay	SRS cognition
	Word delay		Word delay

**Table 9:** Set of discriminant features by clustering result for no node reassignment

VAT k=2	Integrity k=5	Tenacity k=5
CBCL externalizing T score	ABC-Inappropriate speech)	ABC-Inappropriate speech)
BAPQ Avg (Mother)	ADOS social affect	ADOS communication & social
Regression	BAPQ Avg (Mother)	ADI-R Q30 (Overall Level of Language)
Verbal IQ	ADI-R Q30 (Overall level of language)	Regression
	Regression	Social (ADI-R A)
	SRS T score	Word delay
	Verbal score (ADI-R B)	
	Word delay	

## Discussion

Regarding appropriate graph representation, the results confirmed advantageous aspects of the min-conn setting as the kNN2 graph exhibited optimal clusterings that were not sensitive to preprocessing parametric changes compared to the kNN4 graph. This implies robustness of min-connectivity graphs. As expected, there were no significant differences in age and gender distribution across various cluster configurations. This suggests that the variations in the ASD severity is unrelated to age or gender. However, interestingly, the distribution of percentage Caucasian varied across clusters.

We had hypothesized that the results obtained by excluding the critical attack set (i.e. no node reassignment) would result in more clearly defined clusters. This is based on the assumption that the critical attack set contains possible outlier and/or overlapping nodes. As mentioned earlier, outliers in the context of this application could denote patients that may have some errors in their phenotype data from the data collection process. However, the results obtained for the configurations without node reassignment (NR) are not conclusive. The removal of the nodes, though relatively few, impacts the resulting configuration especially for VAT-Clust, which has the largest critical attack set of 108 nodes. When we compare the visualizations (Fig. 1) of the NR results to the traditional clustering results, in which every node is assigned to a cluster, the differences are subtle. This is probably due to the relatively small sizes of the critical attack sets (Table 7) obtained in this work based on the grouping algorithm applied to attain the desired number of clusters. From the statistical analysis (Table 7), the no node reassignment appears beneficial for Tenacity-Clust and Integrity-Clust

The clinical outcomes analyses (Tables 6 and 7) demonstrate the significance and usefulness of the varied cluster configurations. Cluster attributes are consistent in the kNN2 integrity k=3 clustering (Table 6). Cluster C1 has the most severe symptoms by all measures, such as lowest Overall IQ, and highest incidence of epilepsy. Cluster C0 has the lowest overall scores for ABC, RBS-R and ADOS CSS, as well as the highest Vineland Composite Score, Overall IQ, Learning Vocabulary Score (PPVTA), and the lowest incidence of epilepsy. For all measures, Cluster C2 lies between clusters C0 and C1. It is interesting also to note the cluster sizes. For this dataset, the subjects with the most severe symptoms account for approximately 7% of the sample. The group with the least severe symptoms is 71% of the sample, and the middle group counts for 22%. However, the *η*^2^ values are very low which conveys a relatively low confidence in the results.

The clustering obtained by the VAT 4-clustering is in many ways similar to the integrity 3-clustering, as can observed visually by comparing Figs. 1a and c, along with statistical results from Table 6. The most severe cluster has the smallest size while the least severe is the largest cluster. As mentioned in the previous section, the overall effect sizes are consistently high for kNN2 Tenacity 5-Cluster result in Table 6 which conveys a strong confidence in the results. The variations observed in the varying levels of ASD severity complexity is interesting across clusters, for example, between clusters C0 and C4. Cluster C4 is characterized by the largest ASD severity level in terms of low overall IQ, relatively high occurrence epilepsy (non-febrile seizures), low functioning skills (as quantified by the Vineland composite scores), and high ADOS CSS scores. However, their aberrent behavior checklist and stereotyped behavior scores are not the most severe scores. It is slightly better compared to cluster CO. Cluster C0 has very high mean IQ scores (not the highest - C2) but the aberrent behavior checklist and stereotyped Behavior scores for that subgroup is the lowest. This provides further evidence that there is an ASD subgroup with relatively IQ scores but very severe behavioral problems (Obafemi-Ajayi et al. 2015).

**Fig. 4:**
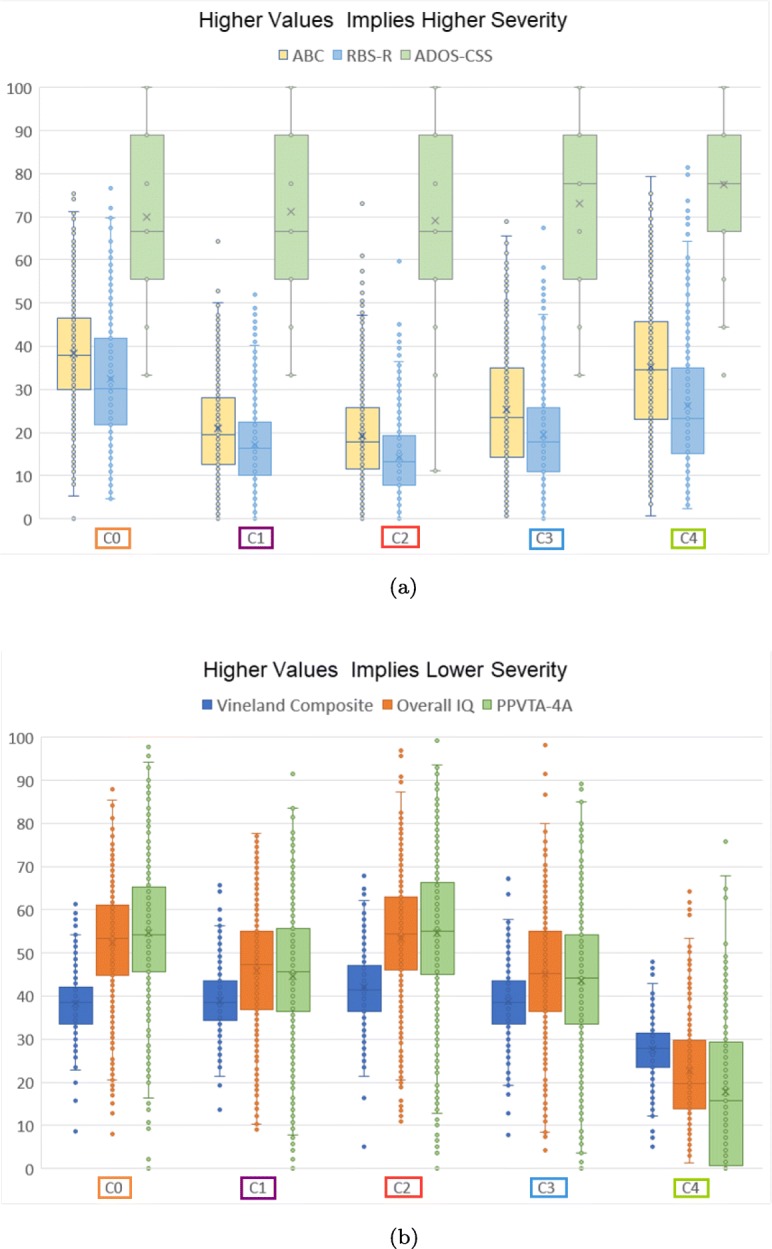
Analysis of ASD outcome measures (normalized values using known features ranges) across clusters for kNN2 Tenacity 5 clustering configuration. The color of the boxes correlate to the colors of the clusters in Fig. 1e. Gold denotes C0, Purple: C1, Red: C2, Blue: C3, and Green: C4. **a** Outcome Measures for which values are positively correlated with ASD severity. **b** Outcome Measures for which values are inversely correlated with ASD severity

Four of the seven optimal clusterings consisted of 5 clusters. Two of the clusterings were obtained using integrity (both with and without reassigment) and two of the clusterings were obtained using tenacity (again both with and without reassignment). These clusterings can be compared visually in Figs. 1 and 2. We can observe that they all share some similarities in their configuration. According to Table 6, the kNN2 Tenacity 5-clustering configuration obtained using the filtered 33 features set had consistently high eta-squared values across all the outcome measures. Figure 4 summarizes the trends across the outcome measures for its five clusters using box plot charts. These charts (Fig. 4) were generated using the normalized values of the outcome measures between 0 and 100 to aid ease of comparisons across the diverse ranges for each measure. The outcome measures for which higher values implies higher ASD severity (ABC, RBS-R and ADOS-CSS) are illustrated in Fig. 4a while the measures for which higher values implies lower ASD severity (ABC, RBS-R and ADOS-CSS) are illustrated in Fig. 4b. Cluster C2 (the red cluster in Fig. 1e) denotes the subgroup with the lowest ASD severity (i.e. high functioning group) across all six measures. It is also the largest subgroup. Cluster C4 (the green cluster in Fig. 1e) denotes the subgroup with the highest ASD severity (i.e. low functioning group) across all six measures. It is also the smallest subgroup. Cluster C0, the gold cluster in Fig. 1e), is characterized by high IQ and PPVTA (vocabulary) scores as well as a low ADOS-CSS score but severe Vineland composite, ABC and RBS-R scores. The RBS-R and ABC scores are the lowest among all the clusters. This suggests that there is a subgroup with high IQ and vocabulary skills but very severe behavioral skills. Cluster C3, the blue cluster in Fig. 1e), is a subgroup that consistently lies in between the C2(least severe, red) and C4 (most severe, green) subgroups in all measures. In contrast, C1, the purple cluster in Fig. 1e), is consistently in between C0 (gold) and C2 (red) except for its ADOS-CSS scores, that is slightly higher for both. When we comparing C1 and C3 subgroups with each other, we can observe that C1 (purple) is less severe than C3 (blue) across all six outcome measures.

The feature extraction results seem to suggest that the following phenotypes could be useful biomarkers in delineating ASD subgroups: Regression, Word Delay, ADI-R Q30 (Overall Level of Language), ADI-R Q86 (Abnormality evidence), RBS-R aggregate score (Ritualistic Behavior), ABC aggregate scores (Irritability, Inappropriate Speech), CBCL Externalizing T Score, Verbal score (ADI-R B), RBS-R-Stereotyped Behavior, BAPQ Avg (Mother), ADI-R C (Repetitive Behavior), Social (ADI-R A), and SRS aggregate scores (Mannerisms, Cognition, overall T Score). These results support evidence that language delay, regression and social scores are useful biomarkers for delineating meaningful subgroups.

## Conclusion

This paper investigated the application of the NBR-Clust graph-based method to cluster analysis of ASD phenotypes of 2680 simplex ASD probands using different node resilience measures. To determine the optimal clustering configuration, we applied a holistic approach using three main criteria: internal cluster validation indices, graph quality measures, and distribution of resulting clusters. We presented a rigorous clinical/behavioral analysis of the highly ranked results by graph type and resilience measure. The results obtained demonstrate the potential and usefulness of NBR-Clust. The results favored a 5-cluster ASD sub-grouping configuration and identified a set of potentially useful phenotype biomarkers. Future work will include refinement of the critical attack set to identify specifically the outlier nodes for enhanced biomarker detection. Further studies are also needed to verify the potential ASD biomarkers identified in this work with respect to their application in management of ASD.

## Additional file

Additional file 1 Cohen-d test values for Tables 6 and 7 to evaluate the effect sizes for each pairwise comparison. (XLSX 32 kb)
